# Transient Sulfenic Acids in the Synthesis of Biologically Relevant Products

**DOI:** 10.3390/molecules23051030

**Published:** 2018-04-27

**Authors:** Anna Barattucci, Maria Chiara Aversa, Aurora Mancuso, Tania Maria Grazia Salerno, Paola Bonaccorsi

**Affiliations:** Dipartimento di Scienze Chimiche Biologiche Farmaceutiche ed Ambientali, Università degli Studi di Messina, 98166 Messina ME, Italy; abarattucci@unime.it (A.B.); aversa@unime.it (M.C.A.); mancusoaurora@gmail.com (A.M.); tsalerno@unime.it (T.M.G.S.)

**Keywords:** sulfenic acids, sulfoxides, sulfones, disulfides, alliin derivatives, glycoconjugates, pyrimidine derivatives

## Abstract

Sulfenic acids as small molecules are too unstable to be isolated and their transient nature offers the possibility to involve them in concerted processes that lead to the obtainment of functional groups such as sulfoxides, sulfones, and disulfides. All these functions are present in a number of natural and synthetic drugs and can represent structural motives inducing biologically relevant properties. In this small review the generation and reactions of sulfenic acid bearing naturally occurring residues are described. Carbohydrate and aminoacid-derived sulfenic acids have been used in concerted addition with triple bonds to obtain alliin derivatives and thiosugars in enantiomerically pure form. Glycoconjugates with sulfinyl, sulfonyl, and disulfane functional groups and pyridine-derived disulfides have been obtained from bis- and tris-sulfinyl precursors of sulfenic acids. Small families of such compounds have been subjected to preliminary biological tests. Starting from the evidence that the control of molecular architecture and the presence of suitable functional groups can play a significant role on the exhibition of biological properties, apoptotic effects on malignant cells by glycoconjugates and inhibitory activity against the important human pathogen *S. aureus* by pyrimidine-derived disulfides have been found.

## 1. Introduction

A great number of natural products and synthetic drugs contain sulfur [1,2], many of them being sulfoxides, sulfones, and disulfides. Sulfoxide is one of the most versatile sulfur functional groups. Its chemical structure and polarization, joint with the stereogenicity of the unsymmetrically substituted sulfinyl group, make chiral sulfur-containing compounds have a fundamental role in biology. Moreover, its widespread exploitation in the pharmaceutical industry is still involving organic chemists in searching for the best way to prepare this function in enantiomerically pure form [3]. Examples of sulfoxides in medicinal chemistry include aprikalim, an activator of the potassium channel, oxisurane, an immunosuppressor, and esomeprazole, the (*S*)-enantiomer of omeprazole, which is the most used drug against the symptom of gastric ulcers and one of the world’s most sold pharmaceuticals [4]. Despite the lack of stereogenicity, the sulfonyl group, with its singular electronic and structural features is present in a large number of biologically active molecules, having good relevance in medicinal chemistry [5]. Eletriptan, used for the symptomatic treatment of migraines, the antibacterial dapsone, or bicalutamide, one of the drugs utilized in the fight against of prostate cancer, contain the sulfone moiety [6]. Thanks to the ubiquity of disulfides in biology, the formation of the S-S bond has always intrigued synthetic organic chemists [7]. The disulfide group has a prominent role in biology and medicine because of its ability to stabilize the folded form of proteins and also because it exhibits a series of valuable bioactivities such as antitumor, antibiotic, and enzyme-inhibitory activities [8]. Cystine and glutathione-disulfide are two examples of molecules containing S-S moiety, the disulfide lipoic acid is used as a dietary supplement and antioxidant [9]. Diallyl disulfide, coming from the alliin decomposition in garlic once cut, shows antimicrobial, insecticidal, and larvicidal properties and seems to have a role in the prevention of colorectal cancer [10].

**Scheme 1:**
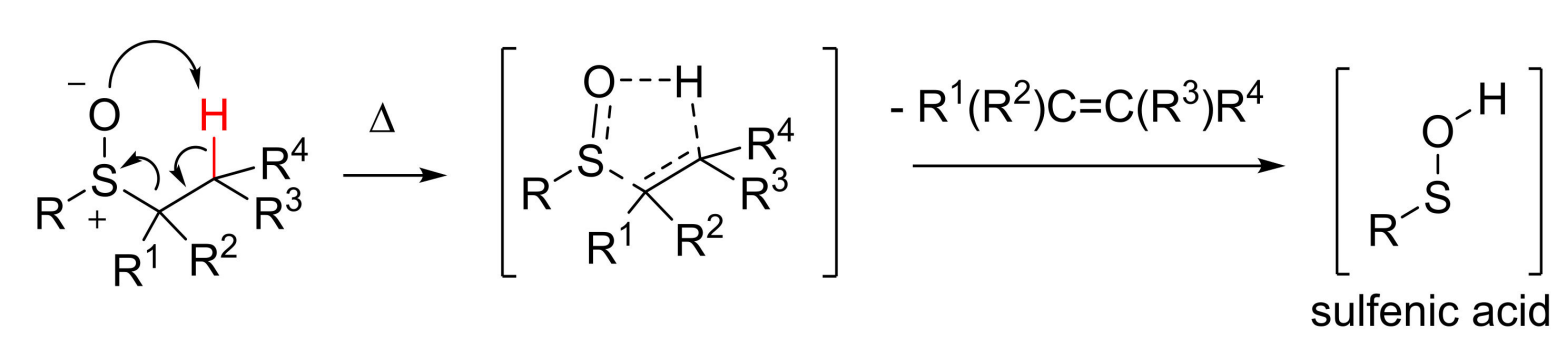
Generation of transient sulfenic acids.

The importance of sulfenic acids RSOH (Scheme 1) as transient intermediates in biological processes is widely recognized. They are usually very reactive intermediates and, in a proteic environment, can be considered as the oxidation product of cysteine thiol moiety [11]. From a synthetic point of view, they can be regarded as useful intermediates in the formation of biologically relevant functions, such as those that have just been cited. Except for a few cases [12], sulfenic acids and the selenenic analogues [13,14] are unstable intermediate species and, among the several ways to obtain sulfenic acids [15,16], their in situ generation via the thermolysis of suitable sulfoxides (Scheme 1) is one of the most exploited methods. The reaction is a *syn* concerted elimination, and passes through a five membered cyclic transition state.

**Scheme 2:**
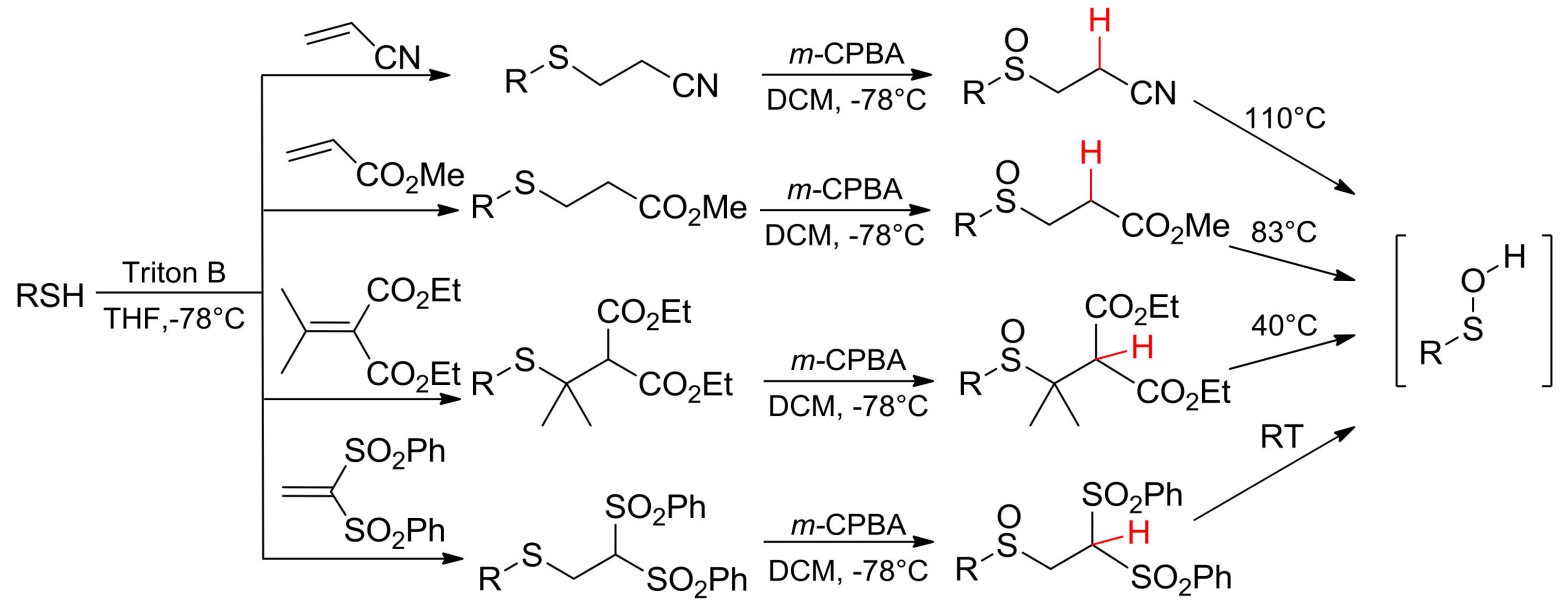
Synthetic route to sulfoxide precursors and their thermolysis temperature.

The synthetic route to the sulfinyl precursors of sulfenic acids generally involvesthe following two steps: the nucleophilic conjugated addition of a thiolate to an α,β-unsaturated compound, and the subsequent oxidation of the obtained thioether to sulfoxide by means of *m-*CPBA. Electron-withdrawing groups linked to the β carbon atom and/or sterically demanding groups on the α carbon atom are needed to enhance the mobility of the β-H and, hence, to modulate the thermolysis temperature (Scheme 2) [17,18]. Tuning of the thermolysis mildness and lack of acidic or basic conditions allow for the generation of sulfenic acids bearing labile R residue, not obtainable in different processes.

**Scheme 3:**
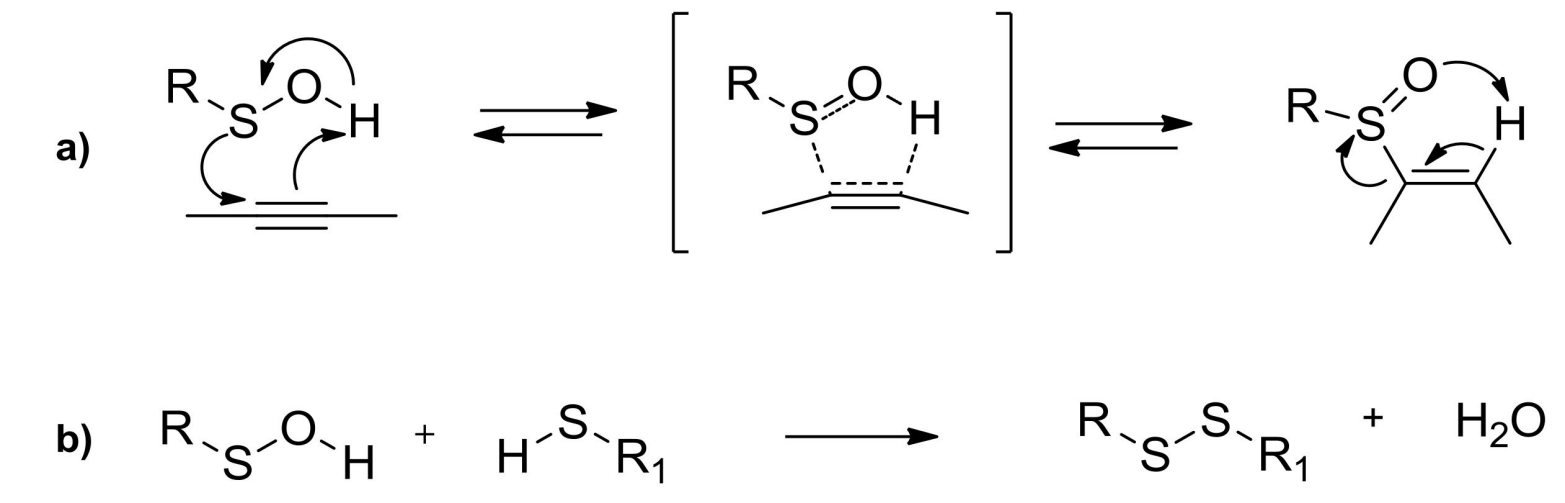
Sulfoxides and disulfides from sulfenic acids.

The most typical and exploited reaction of sulfenic acids is their regioselective concerted *syn-*addition to double or triple bonds, used to create alkyl and vinyl sulfoxides (**a** in Scheme 3). The reaction is highly regioselective [19], depending on the nature of the triple or double bonds. Furthermore, the stereochemical feature of the R group linked at the sulfenic moiety causes a high degree of stereoselection in the new chiral sulfoxides formation. During the last decades, this approach led to the stereoselective synthesis of many kinds of sulfoxides [20], having a large spread of applications. Enantiopure sulfinyl homo- and heterodienes, coming from the approach of chiral sulfenic acid on different kind of alkynes, were involved in highly stereoselective Diels-Alder cycloadditions [21]. The thermolysis of suitable di- and trisulfoxides, with the subsequent in situ generation of di- and trisulfenic acids and their approach to differently functionalized triple bonds, was the key step for the obtainment of multi-armed sulfoxides, which are useful in coordination and supramolecular chemistry, and for the stereoselective formation of sulfinyl and sulfonyl macrocycles [22]. Among others [23], condensation of sulfenic acids with thiols, employed until the last decade only to demonstrate indirectly the in situ generation of these unstable intermediates, has found its application in the formation of disulfide bonds (**b** in Scheme 3). The mild and effective synthesis of a wide range of disulfides bearing two different R groups at the disulfane moiety was accessible [24].

In this short review of our work, the syntheses of a variety of biologically relevant sulfoxides, sulfones, and disulfides are reported. The key step of their formation exploits sulfenic acid intermediation and the two following described reactions: addition to carbon-carbon unsaturation and condensation with thiols. The choice of a suitable sulfinyl precursor for each of the sulfenic acids used in the synthesis of such compounds was crucial. The thermolysis temperature and the solvent were two of the important variables in the concerted processes involved in the in situ generation and reaction of the transient intermediates. 

## 2. Alliin Derivatives

**Scheme 4:**
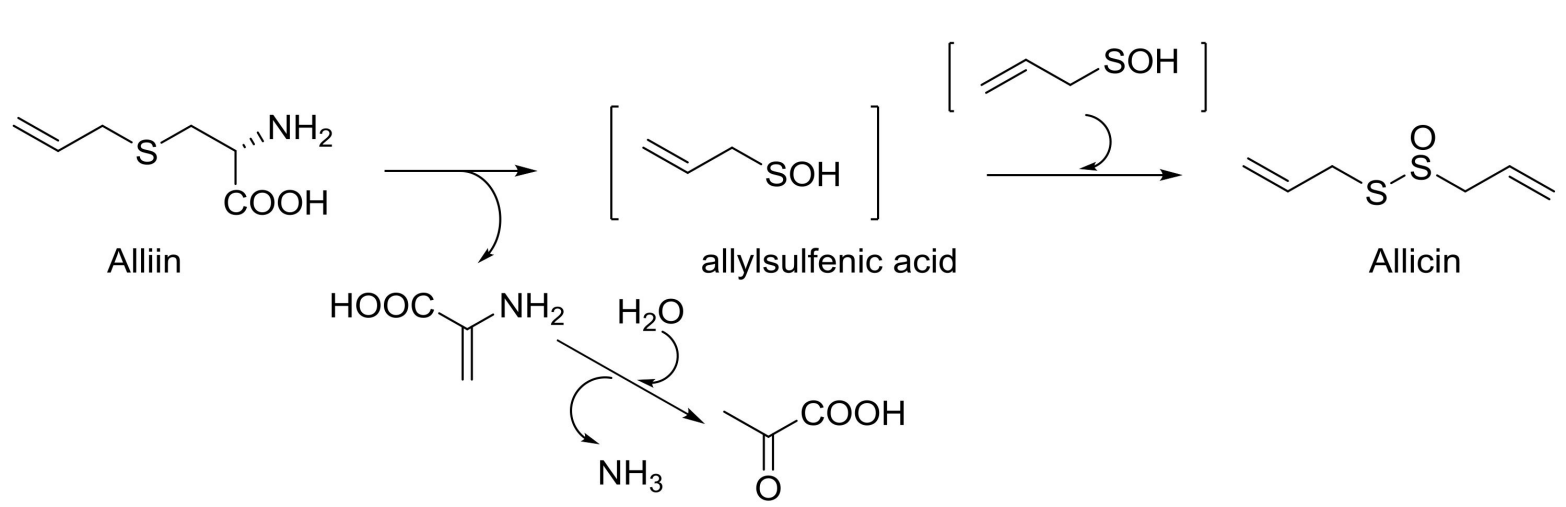
From alliin to allicin, via allylsulfenic acid.

The use of natural products for medicinal purposes is an ancient and still applied practice that joins and completes the process of discovery and development of new drugs in which the pharmaceutical industry is involved. Alliin is a l-cysteine-derived sulfoxide, generated from freshly cut or crushed garlic [10], which has been extensively studied for its anti-inflammatory and antioxidant properties [25] because it lowers blood sugar [26], increases the concentration of insulin in the blood, and recently also because it improves lipogenesis [27]. Based on Granroth’s work [28], S-allilcysteine, generated by glutathione, is oxidized to alliin, then is enzymatically hydrolyzed to produce allylsulfenic acid (Scheme 4). This transient intermediate auto-condenses to allicin, a thiosulfinate with many different biological properties [29], responsible for the typical smell and taste of garlic.

**Scheme 5:**
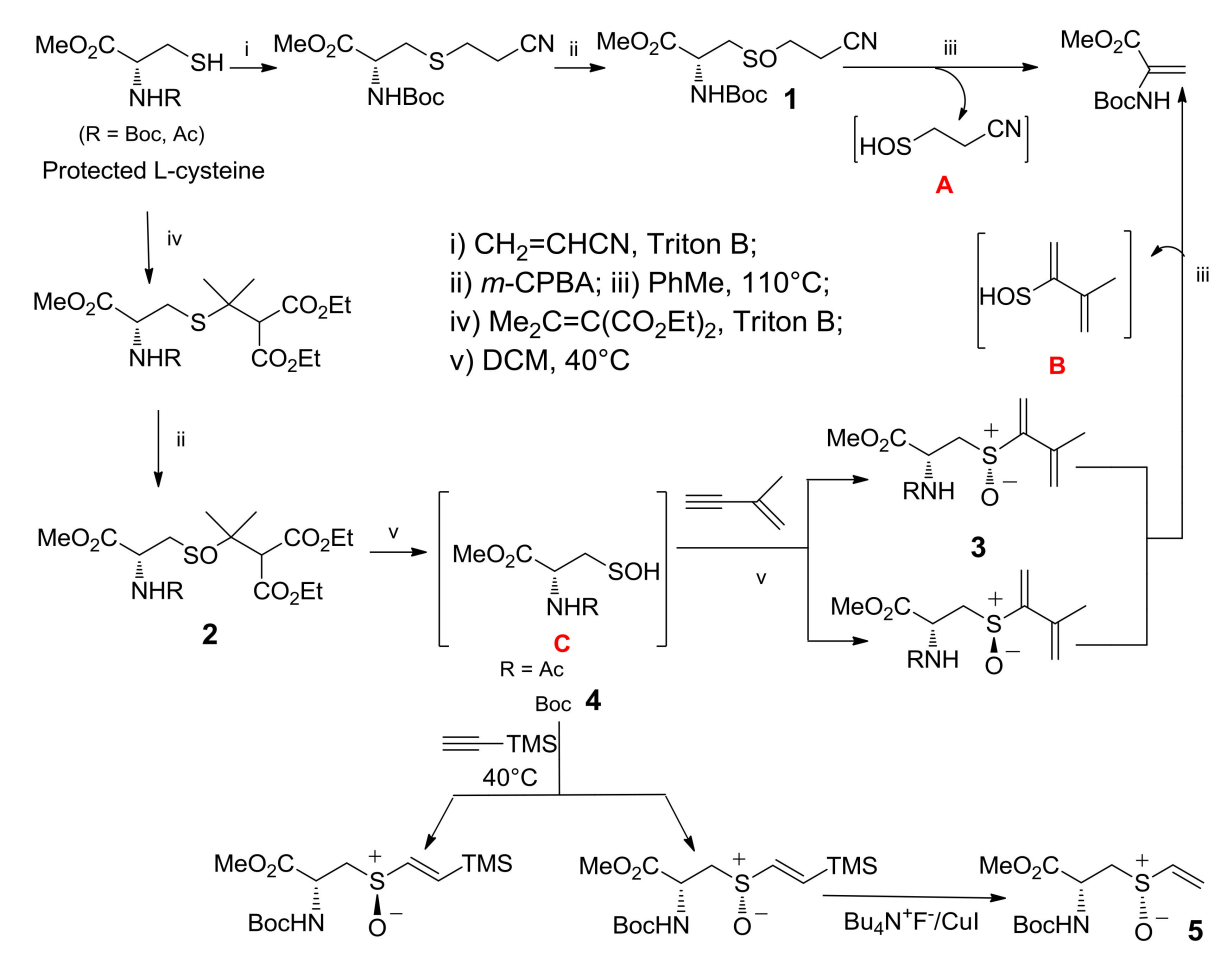
Synthetic pathways for in situ generating l-cysteine-derived sulfenic acids **A**, **B**, and **C**.

In Scheme 5, the stereoselective synthesis, via l-cysteine sulfenic acid **4**, of a derivative of alliin, the *nor*-alliin **5**, is shown. The special features of sulfoxides **1** and **2**, obtained as already described from l-Cysteine, allowed entry to different sulfenic acids [30]. Thermolysis of compound **1** led to the elimination of sulfenic acid **A** due to the following various factors: the competition of two Beta-H to the sulfinyl moiety in the elimination process, the presence of two electron-withdrawing groups on the left-side H atom, and the thermolysis temperature of 110 °C. The structural features of sulfoxide **2** allowed its thermolysis at 40 °C and the generation of the enantiopure *N*-protected l-cysteine sulfenic acid **C**. In particular, *N*-Boc l-Cysteine sulfenic acid **4** was submitted to a concerted addition with trimethylsilyl (TMS)-substituted acetylene, 9 and the addition product was easily transformed into enantiopure *N*-Boc *nor*-alliin **5**. It should be noted that sulfoxides **3**, obtained from the addition of sulfenic acids **C** into the triple bond of 2-methyl-1-buten-3-yne, could generate sulfenic acids **B** by a further elimination of the sulfenic function in a thermolysis at 110 °C.

l-Cysteine has had confirmed its potentiality in the generation of sulfenic acids and the development of synthetic pathways that lead to the introduction of a sulfoxide function into suitably substituted skeletons and to the stereoselective synthesis of biologically relevant compounds such as alliin derivatives.

## 3. Thiosaccharides

In recent decades, interest on the synthesis of glycosidase inhibitors has increased, mainly because these enzymes play a central role in many chronic diseases and biological processes [31]. Most of the glycosidase inhibitors mimic the structures of mono- or disaccharides and thiosugars have been recognized as one of the most popular classes of such inhibitors, with significant therapeutic potential [32]. Some of them also exhibit anti-diabetic activity [33] and some others are used in chemotherapy [34].

Thiosugars are a class of compounds in which the oxygen atom of the sugar ring is replaced with a sulfur atom or has a disaccharide linked via a sulfur bridge. One of the advantages of these compounds over other carbohydrate analogues is their improved oral bioavailability.

**Scheme 6:**
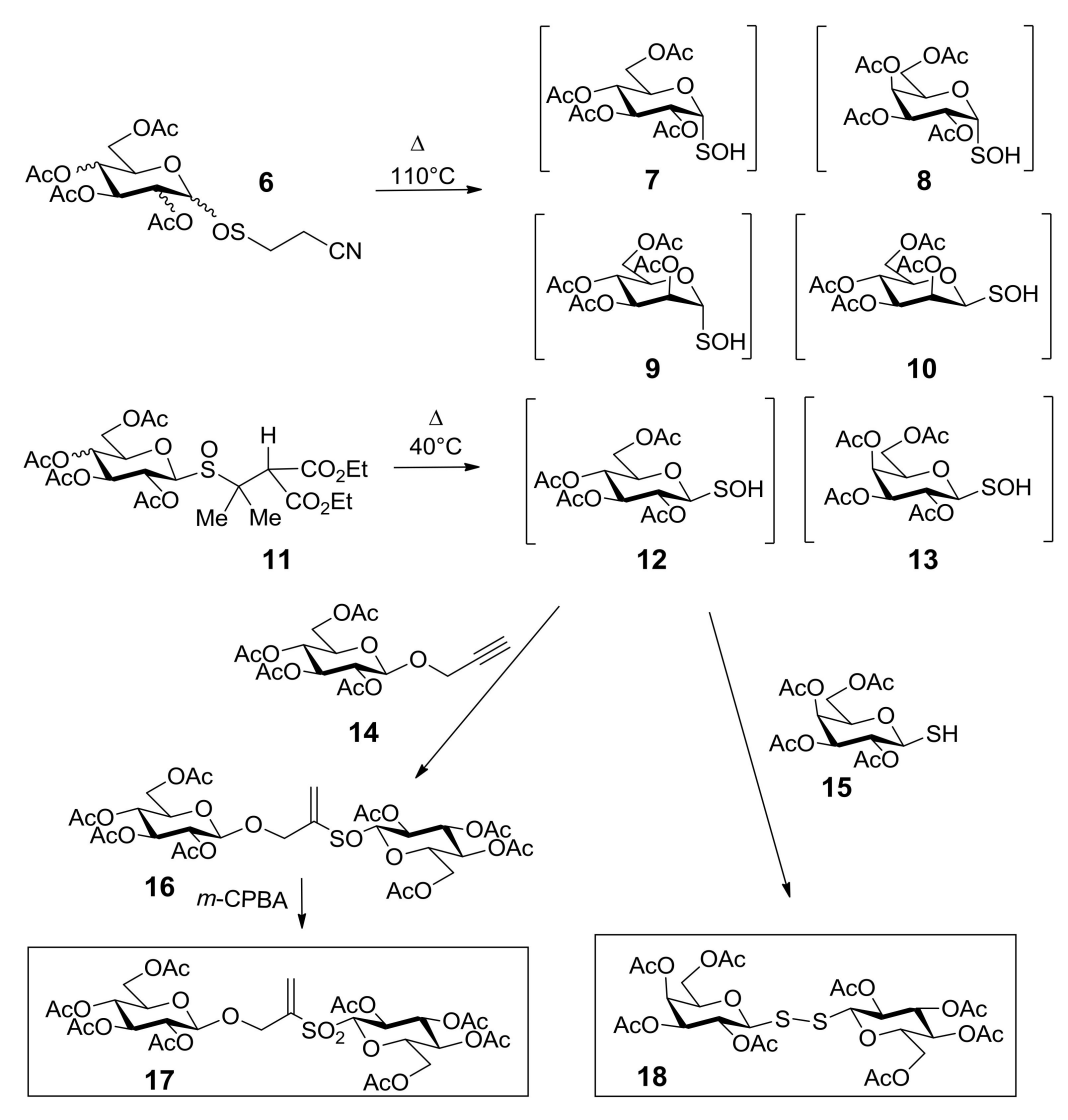
The synthetic pathways to thiosaccharides **17** and **18**.

Sulfoxides **6** and **11** were chosen as the precursors of glycosulfenic acids **7**–**10**, **12**, and **13** [35,36], giving access to transient intermediates that can be easily transformed into thiosaccharides (Scheme 6). In Scheme 6 the synthetic pathways for the synthesis of thiosaccharides **17** and **18** are shown. Compound **18** was obtained by thermolysis at 40 °C of sulfoxide **11** in DCM, in the presence of β-glucopyranoside **14** [24]. The concerted addition of the in situ generated glucosulfenic acid **12** into the triple bond of compound **14** afforded an epimeric mixture of thiosaccharides **16**, which was oxidized to the unique enantiomerically pure thiosaccharide **17**. The condensation of glucosulfenic acid **12** with 1-thiogalactopyranose (**15**) led to thiosaccharide **18**. The disulfide bridge has recently been introduced into carbohydrate chemistry as a new interglycosidic linker [37] that offers some advantages in terms of flexibility, increased distance between the two glycosidic components, and the opportunity to vary the monosaccharide units.

Mild reaction conditions, easy application to a number of monosaccharides, good yields, and retention of configuration at the anomeric carbon atoms of glucosyl derivatives confirmed the potentiality of such original synthetic strategies for the preparation of thiosugars, based on the generation and in situ reaction of glycosulfenic acids.

## 4. Glycoconjugated Disulfoxides and Disulfides

The generation of two or three sulfenic functions [22] in one skeleton opened the way toward the synthesis of tailored small molecules containing two or three glycopyranosyl units separated by alkyl and aryl spacers and the study of the relationship between molecular structure and biological activity of such molecules, some of which exhibited unexpected significant results.

To be successful in the formation of more than one sulfenic function in a single substrate was not at all obvious because of the contemporary oxidation of the sulfidic functions into the sulfinyl precursors of sulfenic acids, avoiding the over-oxidation of even one of the sulfidic functions into sulfone. Very good results were obtained by using sulfinyl precursors that thermolyzed at 40 °C and 110 °C (see Scheme 3 in Section 1). However, the most convenient procedure, for the generation of more than one sulfenic function in a single substrate, involved the use of sulfinyl precursors thermolyzing at 83 °C, for their stability and reactivity, producing final products in better total yields. Moreover, recent experimental observations led us to conclude that the formation of the two or three sulfenic functions is a stepwise process [38,39] that proceeds with the generation of the first sulfenic function and its addition or condensation to suitable molecules, followed by generation of the second sulfenic function and its reaction, and so forth (Scheme 9 in Section 5).

Efforts were made to design molecules with carbohydrates connected to each other by different kspacers to realize small glycoconjugates and study their effects on the cell growth. Glycoconjugates constitute fundamental tools in the discovery process of new therapeutic agents and in the research of information about complex biological processes, such as recognition events [40]. This is because cells are covered by carbohydrates, which are capable of carrying specific biological messages in fundamental processes that regulate cellular growth and death.

**Scheme 7:**
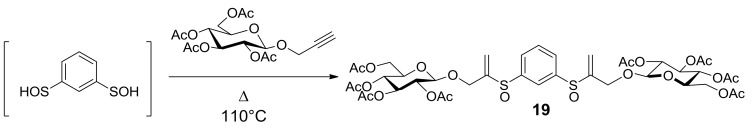
From bis-sulfenic acid to glycoconjugate **19**.

**Figure 1:**
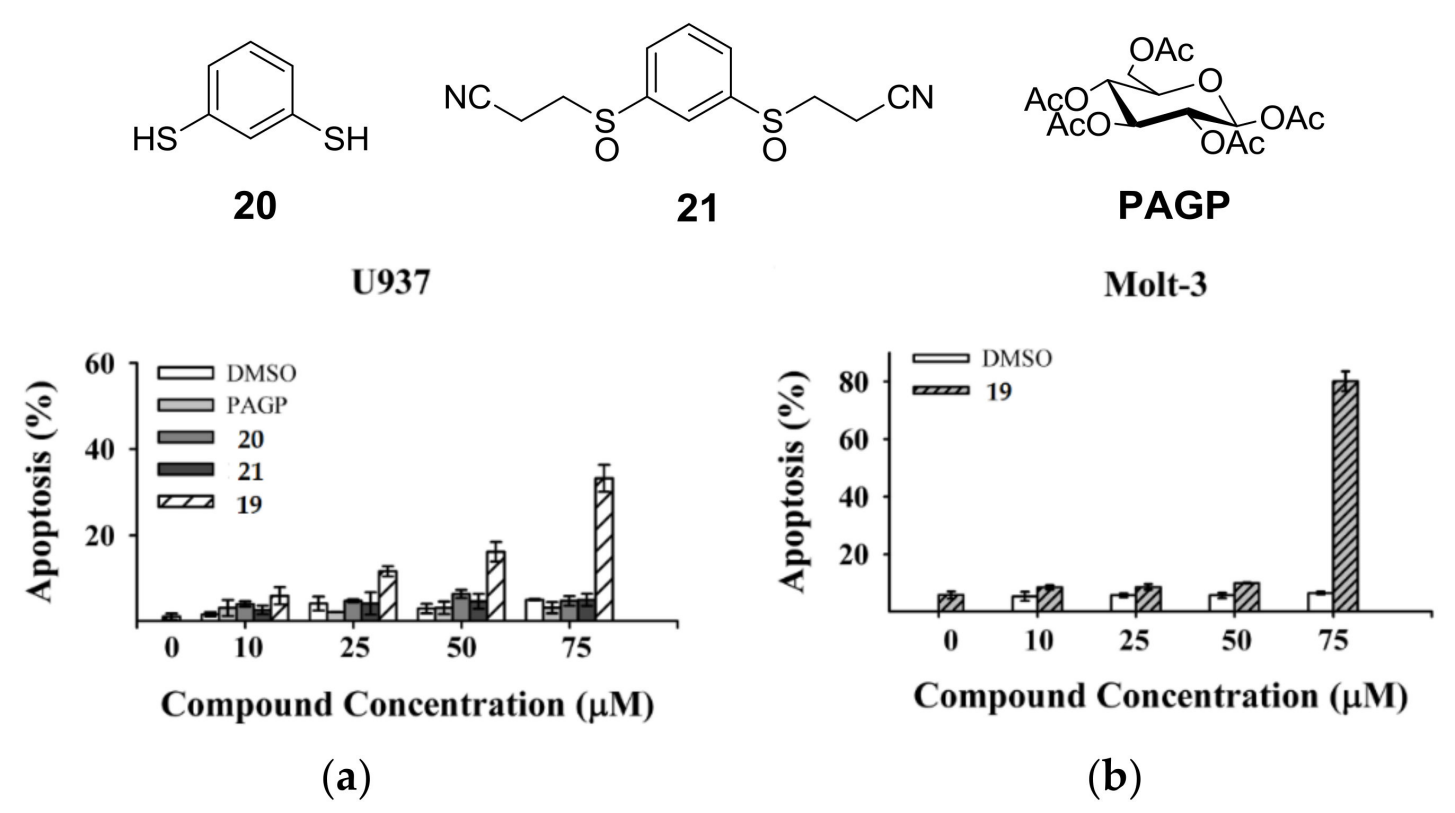
Effect of compound **19** on apoptosis: (**a**) U937 cells were exposed for 24 h to different concentrations of control vehicle (DMSO), PAGP, compounds **19**–**21**. Apoptosis was evaluated by fluorescence microscopy after staining of the cells with acridine orange; (**b**) Molt-3 cells were exposed for 24 h to different concentrations of control vehicle (DMSO) and compound **19**. Apoptosis was evaluated as described in (**a**).

Research started with the synthesis of a small library of thioglycoconjugates in which two glucose units were linked by alkyl and aryl spacers. Among the obtained molecules, sulfinyl bis-β-d-glucopyranoside **19**, shown in Scheme 7, exerted significant effects on cell growth and apoptosis [41]. A trypan blue exclusion test and MTS assay in the monocytic cell line U937 and in the lymphocytic cell line Molt-3 showed that thioglycoconjugate **19** significantly stimulated the growth of both U937 and Molt-3 cells at concentrations ranging from 25 to 50 μM, while the concentration of 75 μM caused a dramatic decrease of total viable cell number. To investigate whether cell toxicity exerted by **19** could be attributable to its capability of inducing apoptotic death, U937 and Molt-3 cells were treated either with the vehicle alone or with **19** at concentrations ranging from 10 to 75 μM and the results were evaluated by microscopy analysis, followed by staining with acridine orange (Figure 1a,b). Compounds **20**, **21**, and PAGP were also analyzed to understand the influence that different significant moieties of **19** could exert on its cytotoxic potential. 1,3-Benzenedithiol (**20**) corresponds to the aromatic central ring, 3,3′-[1,3-phenylenebis(sulfinyl)]bis-propanenitrile (**21**) mimics the rigid core with flexible arms, both constituting the sugar moiety molecule of **19** in its whole. After 24 h incubation, at the lower concentration of 10 μM, compound **19** was found to induce a significantly higher level of apoptosis, in a dose-response fashion, in comparison with the vehicle alone in U937 cells as well as in Molt-3 cells. No significant difference was observed between apoptosis values detected in samples treated with **20**, **21**, or PAGP (Figure 1a,b). These results suggested that apoptosis is the main form of cell death associated with the cytotoxic potential of compound **19** and that the specific induction of apoptosis is a consequence of the interactions of the whole molecular structure of the glycoconjugate **19,** rather than the effect of one of the significant moieties that make it up.

**Figure 2:**
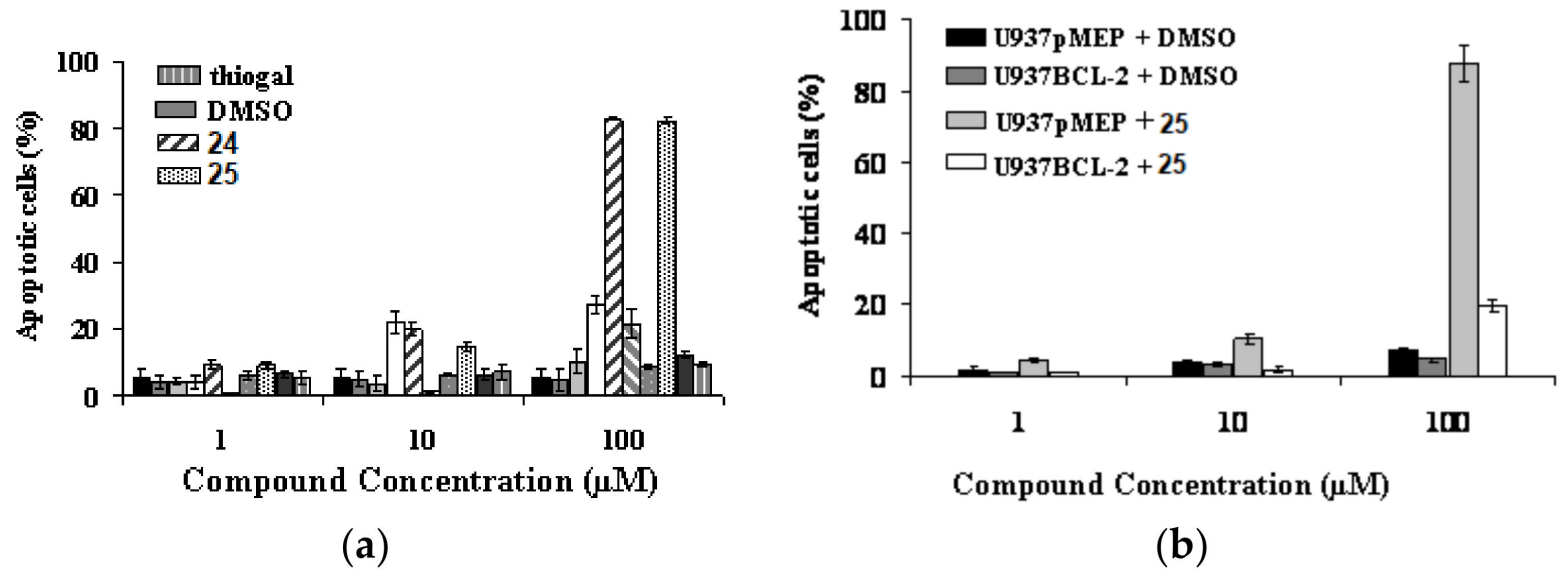
(**a**) Effects of bis(disulfides) **24** and **25** and thiogal on the cytotoxicity in U937 cells after 24 h incubation, in comparison with other molecules of the same family. Percentage nuclei showing apoptotic features at microscopy analysis following staining with acridine orange; (**b**) Effects of bis(disulfide) **25** on apoptosis in U937 transfectants overexpressing Bcl-2 (U937mBCL2) and in U937 control transfectants (U937pMEP), after 24 h incubation.

**Scheme 8:**
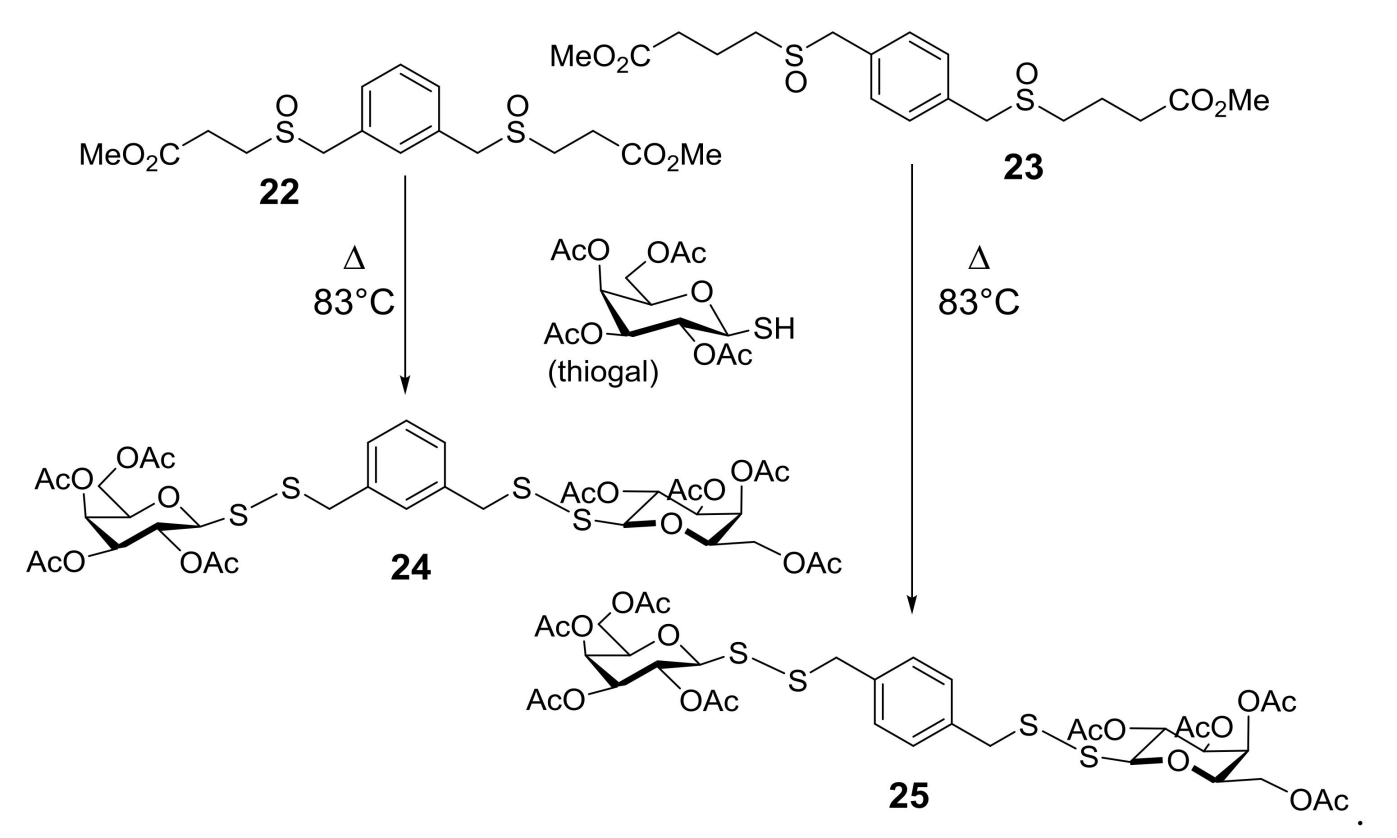
From bis-sulfinyl **22** and **23** precursors of sulfenic acids to glycoconjugates **24** and **25**.

Starting from the biological evidence that the control of molecular architecture and the saccharide spacing could play a significant role on the enhancement of apoptotic properties, a new collection of thioglycoconjugates was synthesized and the behavior of two of them in various cell hlines was examined. Compounds **24** and **25**, in which a benzene ring is linked to two sugar units by disulfide bonds and the monosaccharides are galactose derivatives, were obtained from thermolysis of **22** and **23**, respectively, with 1-thiogalactose (Scheme 8) [42]. The significant and comparable effect of apoptotic death on U937 cells caused by **24** and **25,** and shown in Figure 2a, was further characterized by investigating the expression impact of the anti-apoptotic Bcl-2 protein on apoptosis induced by bis-disulfide **25**. After 24 h treatment of U937cells, transfectants overexpressing Bcl-2 (U93 BCL2) and U937 controlled transfectants (U937pMEP) with compound **25**, it was quite clear that over-expression of Bcl-2 highly, even if not fully, protected U937mBCL2 cells from apoptosis induced by bis-disulfide **25**. Conversely, U937pMEP cells were highly sensitive, as expected, to apoptosis (Figure 2b). These experiments indicated that apoptosis induced in U937 cells by this new class of compounds corresponds to the classical, Bcl-2-sensitive, mitochondrial-dependent, intrinsic form of apoptotic cell death. Incidentally, the effect of inducing apoptotic the cell death of compounds **24** and **25** was extended to a panel of human cancer cell lines, some of which are considered as very aggressive and resistant to chemotherapeutic agents.

**Figure 3:**
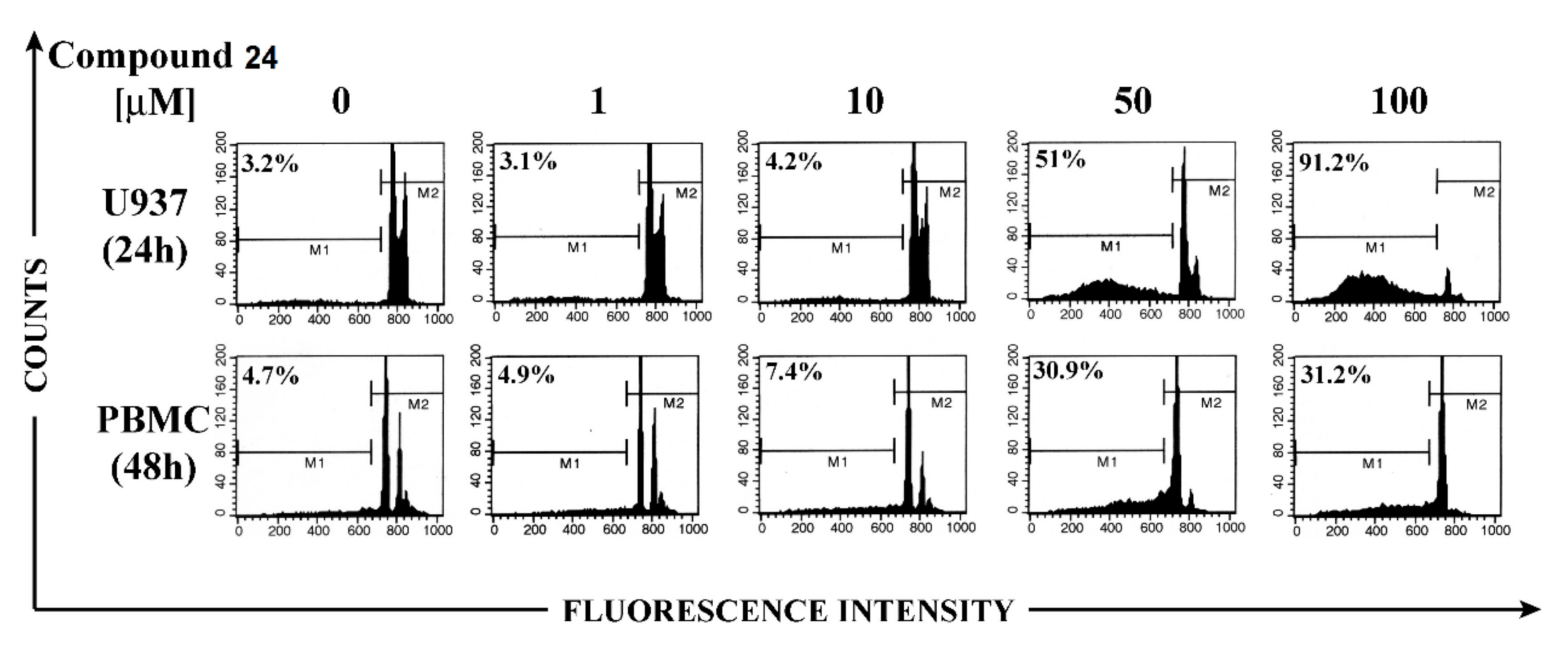
Effect of compound **24** on apoptosis in U937 cells and in peripheral blood mononuclear cells (PBMC). U937 cells and PBMC were treated with 0, 1, 10, 50, and 100 μM of compound **24** for 24 and 48 h, respectively. Apoptosis was evaluated by hypodiploid nuclei analysis after DNA staining with propidium iodide. Percentages of hypodiploid nuclei (M1) are reported in the cytograms.

Finally, the apoptotic effect of bis-disulfide **24** on U937 cells was compared with that exerted on non-malignant cells, using peripheral blood mononuclear cells (PBMCs) from healthy donors and flow cytometry analysis, following propidium iodide staining to detect hypodiploid apoptotic nuclei. Figure 3 shows that levels of apoptosis detected on PBMCs were appreciable only after 48 h incubation at the high concentrations and even in this case they were significantly lower than those observed in U937 cells.

The benzene platform, accommodating two flexible sulfureted arms that connect two glycosyl units, has shown to be a suitable skeleton for molecules that have specific cytotoxic activity against several tumoral cell lines. In particular, galactoconjugates exerted the most significant effects, differentiating between cancer and healthy cells. Even in these cases the pathways for their synthesis are based on the generation of sulfenic acids and their ability to turn into sulfureted groups such as sulfoxides, sulfones, and disulfides, all contributing to the appearance of the observed biological activity.

## 5. Pyrimidine-Derived Disulfides

The request for new compounds endowed with antimicrobial activity [43] that show, possibly, new mechanisms of action, has become a necessity to curb the worldwide problem of antibiotic resistance. *Staphylococcus aureus* (*S. aureus*) is perhaps the pathogen of greatest concern because of its intrinsic virulence and its ability to develop exactly the above-cited resistance [44].

Several derivatives of pyrimidine have been employed in the synthesis of compounds with notable antimicrobial activity [45]. Therefore, the sulfenic acid/thiol condensation was used to prepare a small collection of mono-, di-, and tri-pyrimidine-derived disulfides [46] to be subjected to a biological screening against various pathogens.

**Scheme 9:**
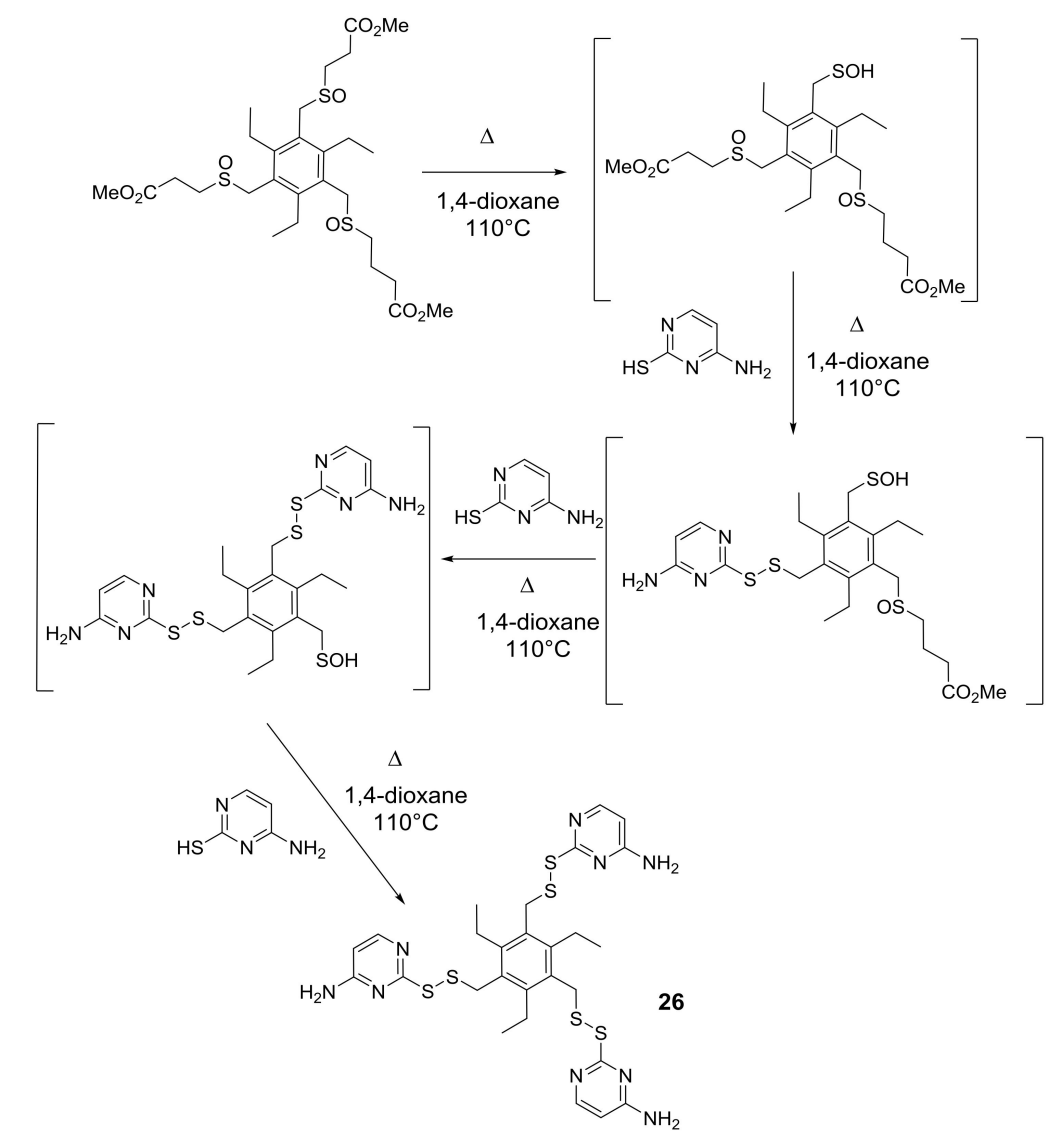
From tris-sulfinyl precursors of sulfenic acids to pyrimidine-derived tris-disulfide 26.

In Scheme 9 the stepwise formation of the three sulfenic functions, which in turn condensed to thiocytosine leading to compound **26**, is shown as example. Although the chosen sulfinyl precursor thermolyzes at 83 °C (Scheme 1 in Section 1), side-products, due to the nucleophilic attack of the sulfur atom of thiocytosine to electron-poor carbon atoms of DCE, lowered the yield of final product 26 and the other pyrimidine-derived molecules. In order to avoid this side-reaction, several other solvents were tested, such as acetonitrile, THF, or 1,4-dioxane (bp 101 °C) and the last one resulted in the most efficient, in terms of yield and work-up of the thermolysis crude.

An introductory study on the antimicrobial activity of the family of pyrimidine-derived compounds, the family compound **26** belongs to, was undertaken just after having ascertained that all of them revealed no cytotoxicity against human erythrocytes [47]. In particular, the trypan blue assay showed a viability of 92–100% in all experiments and hemolytic activity was determined by hemoglobin release in the plasma after exposure to the molecules. The in vitro antibacterial and antifungal activity was then investigated against representative Gram-negative (*Escherichia coli* ATCC 25922, *Pseudomonas aeruginosa* ATCC 27853), Gram-positive (*Bacillus cereus* ATCC 11778, *S. aureus* ATCC 29213), and yeast (*Candida albicans* ATCC 10231, *Cryptococcus neoformans* ATCC MYA-565) strains. The bacterial MIC of all compounds was determined in vitro using the broth dilution method in Mueller Hinton medium. A total inhibition of growth of the examined Gram-positive *S. aureus* strains was observed at concentrations of 50 μg/mL to 25 μg/mL for tris-disulfide 26 and two other molecules of the same family. No others of the investigated pathogen species showed growth inhibition effects after treatment with the bis- and tris-disulfides under study. In addition, no antifungal effect was observed for the two pathogenic fungi included in this study, indicating that these pyrimidine-derived disulfides can be opportunely modified to enhance a species-specific inhibitory activity against the important human pathogen *S. aureus*.

## 6. Conclusions

The chemistry that can be found in this paper is more the development of the use of sulfenic acids in the synthesis of biologically interesting targets, than the development of new therapeutic molecules. The process of development for new medicinal targets is a long and difficult way to obtain compounds that have powerful effects on certain pathologies that affect human life, with the fewest possible side effects. It starts with the discovery of molecules that possess chemical features associated with a specific activity and the first step of the discovery process is generally entrusted to chemists. They take inspiration from nature, looking at the following two important aspects of molecules: structure and functional groups. Indeed, the sulfenic moiety has been studied, by chemists and biologists, for its antioxidant activity, its part in signal transduction and transcription regulation events in cells, and for its catalytic and structural roles in enzymes. Its intrinsic transient nature, except in biological systems, does not allow it to be a stable functional group but promotes its ability to turn into a number of biologically significant functionalities. With this paper, focused on the potentiality of sulfenic acids in the synthesis of target molecules, our intent was to show how we have used the chemistry of such transient species in the synthesis of sulfoxide, sulfone, and disulfide functionalities. All of them play a significant role in molecules that are potential medicinal targets or are still natural and synthetic drugs. We have focused our attention on the generation of sulfenic acids bearing natural residues such as amino acids and carbohydrates, with the idea of transforming them into target molecules with structures and functionalities that could exhibit significant biological activities. The synthesis of small families of glycoconjugated sulfoxides, disulfides, and pyrimidine-based disulfides represents the first step in the process of developing new medicinal targets, which involves the observation of a specific biological activity connected to a specific structural typology. We have shown how the addition of sulfenic acids to unsaturated molecules can be used not only as a reaction for the trapping and recognition of such transient species but also as a tool to obtain sulfoxide functions, even in an enantiomerically pure form. We have also used the already known condensation of thiols with sulfenic acids to provide an easy access to molecules with a biologically significant disulfide bond linked to two structurally different residues.

The results of our biological tests encourage us to continue searching for ways to improve the specific effects of the new compounds, and the development of the sulfenic acids chemistry pushes us to provide further contributions in this direction.

## Abbreviations

The following abbreviations are used in this manuscript:

DCE	1,2-dichloroethane
DCM	dichloromethane
DMSO	dimethylsulfoxide
MTS	5-[3-(carboxymethoxy)phenyl]-3-(4,5-dimethyl-2-thiazolyl)-2-(4-sulfophenyl)-2*H*-tetrazolium inner salt
PAGP	1,2,3,4,6-penta-*O*-acetyl-β-d-glucopyranose
RT	room temperature
THF	tetrahydrofuran
Triton B	benzyltrimethylammonium hydroxide

## References

[1] Feng M., Tang B., Liang S.H., Jiang X. (2016). Sulfur Containing Scaffolds in Drugs: Synthesis and Application in Medicinal Chemistry. Curr. Top. Med. Chem..

[2] Ilardi E.A., Vitaku E., Njardarson J.T. (2014). Data-Mining for Sulfur and Fluorine: An Evaluation of Pharmaceuticals to Reveal Opportunities for Drug Design and Discovery. J. Med. Chem..

[3] Han J., Soloshonok V.A., Klika K.D., Drabowicz J., Wzorek A. (2018). Chiral sulfoxides: Advances in asymmetric synthesis and problems with the accurate determination of the stereochemical outcome. Chem. Soc. Rev..

[4] Bentley R. (2005). Role of sulfur chirality in the chemical processes of biology. Chem. Soc. Rev..

[5] Liu N.-W., Liang S., Manolikakes G. (2016). Recent Advances in the Synthesis of Sulfones. Synthesis.

[6] Meadows D.C., Gervay-Hague J. (2006). Vinyl sulfones: Synthetic preparations and medicinal chemistry applications. Med. Res. Rev..

[7] Shiri L., Ghorbani-Choghamarani A., Kazemi M. (2016). S–S Bond Formation: Nanocatalysts in the Oxidative Coupling of Thiols. Aust. J. Chem..

[8] Lee M.H., Yang Z., Lim C.W., Lee Y.H., Dongbang S., Kang C., Kim J.S. (2013). Disulfide-Cleavage-Triggered Chemosensors and Their Biological Applications. Chem. Rev..

[9] Rahman I., MacNee W. (2000). Regulation of redox glutathione levels and gene transcription in lung inflammation: Therapeutic approaches. Free Radic. Biol. Med..

[10] Block E. (1992). The Organosulfur Chemistry of the Genus *Allium*—Implications for the Organic Chemistry of Sulfur. Angew. Chem. Int. Ed. Engl..

[11] Gupta V., Carroll K.S. (2014). Sulfenic acid chemistry, detection and cellular lifetime. Biochim. Biophys. Acta.

[12] Goto K., Tokitoh N., Okazaki R. (1995). Synthesis of a Stable Arenesulfenic Acid Bearing a Bowl-Shaped Macrobicyclic Cyclophane Skeleton. Angew. Chem. Int. Ed..

[13] Aversa M.C., Barattucci A., Bonaccorsi P., Temperini A. (2011). Regio- and Stereocontrolled Synthesis of (*Z*)-α-(Phenylseleno)sulfinyl and -sulfonyl Alkenes via Sulfenic Acids, and a Study of their Reactivity. Eur. J. Org. Chem..

[14] Minuti L., Barattucci A., Bonaccorsi P.M., Di Gioia M.L., Leggio A., Siciliano C., Temperini A. (2013). Intramolecular Displacement of Phenylselenone by a Hydroxy Group: Stereoselective Synthesis of 2-Substituted Tetrahydrofurans. Org. Lett..

[15] Aversa M.C., Barattucci A., Bonaccorsi P., Giannetto P. (2007). Recent Advances and Perspectives in the Chemistry of Sulfenic Acids. Curr. Org. Chem..

[16] Pan J., Carroll K.S. (2015). Light-Mediated Sulfenic Acid Generation from Photocaged Cysteine Sulfoxide. Org. Lett..

[17] Adams H., Anderson J.C., Bell R., Jones D.N., Peel M.R., Tomkinson N.C.O. (1998). The synthesis and Diels–Alder reactions of (*E*)- and (*Z*)-1-methoxy-3-(phenylsulfinyl)buta-1,3-dienes. J. Chem. Soc. Perkin Trans. 1.

[18] Aversa M.C., Barattucci A., Bonaccorsi P., Rollin P., Tatibouët A. (2009). 2,2-Bis(phenylsulfonyl)ethyl sulfides as efficient precursors of sulfenic acids. Arkivoc.

[19] Aversa M.C., Barattucci A., Bonaccorsi P., Contini A. (2009). Addition of sulfenic acids to monosubstituted acetylenes: A theoretical and experimental study. J. Phys. Org. Chem..

[20] Gelat F., Jayashankaran J., Lohier J.F., Gaumont A.C., Perrio S. (2011). Organocatalytic Asymmetric Synthesis of Sulfoxides from Sulfenic Acid Anions Mediated by a Cinchona-Derived PhaseTransfer Reagent. Org. Lett..

[21] Aversa M.C., Barattucci A., Bonaccorsi P., Faggi C., Gacs-Baitz E., Marrocchi A., Minuti L., Taticchi A. (2005). Maleimide cycloadditions by sulfinyldienes: Is the sulfur configuration the only controller of the diastereofacial selectivity?. Tetrahedron.

[22] Aversa M.C., Barattucci A., Bonaccorsi P., Faggi C., Papalia T. (2007). Thiacyclophane Cages and Related Bi- and Tripodal Molecules via Transient Polysulfenic Acids. J. Org. Chem..

[23] Wu S., Lei X., Fan E., Sun Z. (2018). Thermolysis-Induced Two- or Multicomponent Tandem Reactions Involving Isocyanides and Sulfenic-Acid-Generating Sulfoxides: Access to Diverse Sulfur-Containing Functional Scaffolds. Org. Lett..

[24] Aversa M.C., Barattucci A., Bonaccorsi P. (2009). Efficient Synthesis of Unsymmetrical Disulfides through Sulfenic Acids. Eur. J. Org. Chem..

[25] Gu X., Zhu Y.Z. (2011). Therapeupic applications of organosulfur compounds as novel hydrogen sulfide donors and/or mediators. Expert Rev. Clin. Pharmacol..

[26] Augusti K.T., Sheela C.G. (1996). Antiperoxide effect of S-allyl cysteine sulfoxide, an insulin secretagogue, in diabetic rats. Experientia.

[27] Lu J., Cheng B., Meng Z., Fang B., Li T., Sun M., Liu M., Guan S. (2018). Alliin attenuates 1,3-dichloro-2-propanol-induced lipogenesis in HepG2 cells through activation of the AMP-activated protein kinase-dependent pathway. Life Sci..

[28] Granroth B. (1970). Biosynthesis and decomposition of cysteine derivatives in onion and other *Allium* species. Ann. Acad. Sci. Fenn. Chem..

[29] Borlinghaus J., Albrecht F., Gruhlke M.C.H., Nwachukwu I.D., Slusarenko A.J. (2014). Allicin: Chemistry and Biological Properties. Molecules.

[30] Aversa M.C., Barattucci A., Bonaccorsi P., Giannetto P. (2005). l-Cysteine, a Versatile Source of Sulfenic Acids. Synthesis of Enantiopure Alliin Analogues. J. Org. Chem..

[31] Brás N.F., Cerqueira N.M., Ramos M.J., Fernandes P.A. (2014). Glycosidase inhibitors: A patent review (2008–2013). Expert Opin. Ther. Pat..

[32] Wadood A., Ghufran M., Khan A., Azam S.S., Jelani M., Uddin R. (2018). Selective glycosidase inhibitors: A patent review (2012-present). Int. J. Biol. Macromol..

[33] Tamura Y., Miyagawa H., Yoshida T., Chuman H. (2015). Binding interaction of SGLT with sugar and thiosugar by the molecular dynamics simulation. Biochim. Biophys. Acta Biomembr..

[34] Witczak Z.J. (1999). Thio Sugars: Biological Relevance as Potential New Therapeutics. Curr. Med. Chem..

[35] Aucagne V., Aversa M.C., Barattucci A., Bonaccorsi P., Giannetto P., Rollin P., Tatibouët A. (2002). Sulfenic Acids in the Carbohydrate Field. Synthesis of Transient Glycosulfenic Acids and Their Addition to Unsaturated Acceptors. J. Org. Chem..

[36] Aversa M.C., Barattucci A., Bilardo M.C., Bonaccorsi P., Giannetto P., Rollin P., Tatibouët A. (2005). Sulfenic Acids in the Carbohydrate Field. An Example of Straightforward Access to Novel Multivalent Thiosaccharides. J. Org. Chem..

[37] Ramström O., Lehn J.-M. (2000). In Situ Generation and Screening of a Dynamic Combinatorial Carbohydrate Library against Concanavalin A. ChemBioChem.

[38] Barattucci A., Di Gioia M.L., Leggio A., Minuti L., Papalia T., Siciliano C., Temperini A., Bonaccorsi P. (2014). Stereoselective Synthesis of Dithia[3.3]cyclophane *S*,*S*′-Dioxides with Planar and Central Chirality. Eur. J. Org. Chem..

[39] Barattucci A., Bonaccorsi P., Papalia T., Manganaro N., Gattuso G. (2014). Kinetic control in the formation of meso-dithia[3.3]-paracyclophane S,S′-dioxide. Tetrahedron Lett..

[40] Galan M.C., Benito-Alifonso D., Watt G.M. (2011). Carbohydrate chemistry in drug discovery. Org. Biomol. Chem..

[41] Aversa M.C., Barattucci A., Bonaccorsi P., Marino-Merlo F., Mastino A., Sciortino M.T. (2009). Synthesis and biological testing of thioalkane- and thioarene-spaced bis-d-glucopyranosides. Bioorg. Med. Chem..

[42] Bonaccorsi P., Marino-Merlo F., Barattucci A., Battaglia G., Papaianni E., Papalia T., Aversa M.C., Mastino A. (2012). Synthesis and biological evaluation of a new class of glycoconjugated disulfides that exhibit potential anticancer properties. Bioorg. Med. Chem..

[43] Siciliano C., Barattucci A., Bonaccorsi P., Di Gioia M.L., Leggio A., Minuti L., Romio E., Temperini A. (2014). Synthesis of d-erythro-Sphinganine through Serine-Derived α-Amino Epoxides. J. Org. Chem..

[44] Kaźmierczak Z., Górski A., Dąbrowska K. (2014). Facing antibiotic resistance: *Staphylococcus aureus* phages as a medical tool. Viruses.

[45] Jubeen F., Iqbal S.Z., Shafiq N., Khan M., Parveen S., Iqbal M., Nazir A. (2018). Eco-friendly synthesis of pyrimidines and its derivatives: A review on broad spectrum bioactive moiety with huge therapeutic profile. Synth. Commun..

[46] Aversa M.C., Barattucci A., Bonaccorsi P. (2011). From Transient Sulfenic Acids to Disulfide-Functionalized Tripodal Structures. Synlett.

[47] Bonaccorsi P., Barattucci A., Papalia T., Criseo G., Faggio C., Romeo O. (2015). Pyrimidine-derived disulfides as potential antimicrobial agents: Synthesis and evaluation in vitro. J. Sulfur Chem..

